# Primary extramedullary plasmacytoma of the eyelid conjunctiva – A case report and review of the literature

**DOI:** 10.1016/j.amsu.2020.04.028

**Published:** 2020-05-07

**Authors:** Khalid M. Alshomar, Sulaiman M. Altariqi, Ammar C. Alrikabi, Hind M. Alkatan, Yasser H. Al-Faky

**Affiliations:** a Ophthalmology Department, College of Medicine, King Saud University, Riyadh, Saudi Arabia; b Pathology Department, College of Medicine, King Saud University, Riyadh, Saudi Arabia

**Keywords:** Plasmacytoma, Conjunctiva, Multiple myeloma, Extramedullary, Plasma cell tumor, Histopathology

## Abstract

Extramedullary plasmacytomas (EMPs) are uncommon plasma cell tumors that develop in soft tissue as isolated tumors without osseous involvement while secondary lesions are associated with systemic multiple myeloma (MM). Primary extramedullary lesions are most commonly found in upper respiratory tract, gastrointestinal tract and lymph nodes. They can be found either in patients with history of MM or preceding the manifestation of a systemic disease. Orbital manifestation of the lesion is rare but conjunctival involvement is very unusual. The reported cases in the English-written literature are only five cases. Herein, we report the sixth case of primary EMP in a middle-aged adult who presented with a lesion confined to the conjunctiva, unremarkable present and past medical history, and confirmed tissue diagnosis. In addition, a review and summary of the previously reported cases in the literature is presented. We aim to attract the attention of ophthalmic surgeons to consider plasmacytoma within the differential diagnosis of a conjunctival lesion.

## Highlights

• Extramedullary plasmacytomas (EMP) are isolated primary soft tissue plasma cell tumors without osseous involvement.
• Secondary lesions are associated with systemic multiple myeloma.
• The conjunctiva is a very unusual location for primary lesions.
• We are reporting the sixth case of conjunctival primary EMP.
• All cases including ours are summarized.

## 1 Introduction

Extramedullary plasmacytomas (EMPs) are classified into primary and secondary lesions. Primary lesions are uncommon plasma cell tumors that develops in soft tissue as isolated tumors without osseous involvement, while secondary lesions are associated with systemic multiple myeloma (MM) [1]. Primary EMPs are most commonly found in upper respiratory tracts. Other sites include gastrointestinal tract, bladder, breast, thyroid and lymph nodes. Orbital involvement is rare and conjunctival involvement is very unusual [2,3]. In this report, we present a middle-aged adult who presented to our academic institution with primary EMP, which was confined to the conjunctiva and was confirmed histopathologically in addition to a summary of the 5 similar reported conjunctival cases in the literature.

## 2 Case report

A 44-year-old Pakistani male, who was not known to have medical illnesses, and walked into our academic institution with a right lower lid inner palpebral swelling for one month. The mass was painless and progressively increasing in size. There was no history of tearing or discharge. The patient reported an unclear history of ocular surface surgery 5 years prior to his presentation for excision of a smaller conjunctival lesion of unknown nature in the same location. No histopathological details about that lesion were available and no diagnosis was obtained from the history. He was not receiving any regular medications and he was not a known smoker. He was not a good historian especially because of language barrier, however limited family history was obtained and was not found to be significant.

**Fig. 1:**
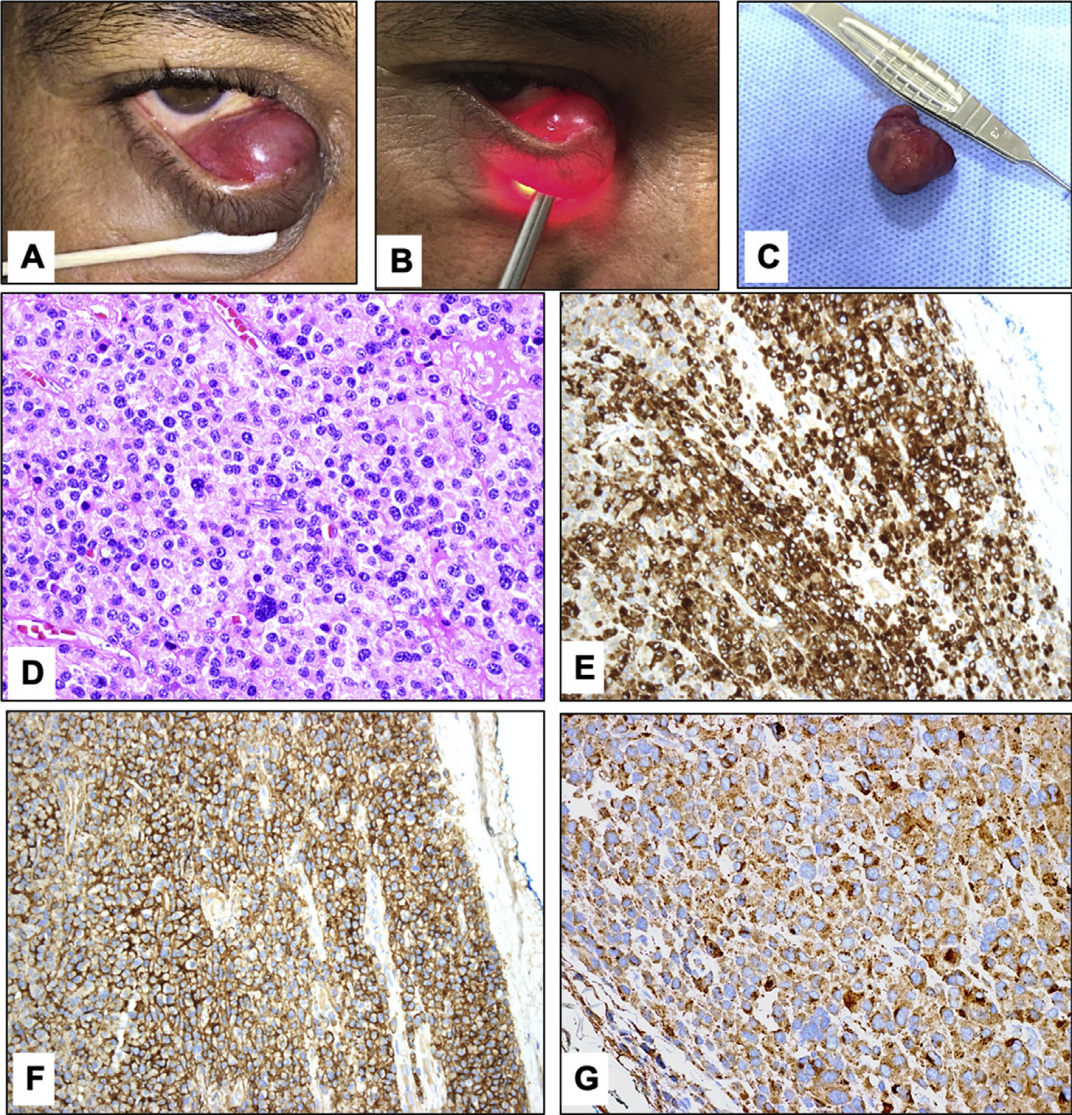
**A:** The clinical appearance of the wee-circumscribed Right lower palpebral conjunctival mass**. B:** The dim light transillumination of the mass. **C:** The mass excised with the impression of cavernous hemangioma measuring 2 × 1.8 × 0.4 cm. **D:** The histopathologic appearance of the proliferating plasma cells and few giant tumor cells within the conjunctival stroma (Original magnification x400 Hematoxylin and eosin). **E and F:** The cells expressing staining for CD79a, and CD138 (Original magnification x200). **G:** The expression for Kappa light chains only by the plasma cells (Original magnification x400).

Upon examination, the visual acuity was 20/20 in both eyes with unremarkable ocular examination. The patient had a right lower lid mass in the medial half of the palpebral conjunctiva measuring 20 mm × 20 mm. The mass was painless, non-tender, reddish in color, soft and semi-cystic in consistency with smooth surface that trans-illuminates but with dim content (Fig. 1a & b). No discharge or bleeding was noted. The right lower punctum was slit-like in shape within the mass. The proposed clinical diagnoses included adult onset xanthogranuloma and other possible cystic lesions such as vascular lesions and epithelial cysts.

The patient was consented for an excisional biopsy of the mass, which was completely excised by an experienced oculoplastic surgeon through conjunctival approach under local anaesthesia within one week of his initial presentation. During excision the lesion was causing remarkable distention of the lower canaliculus without destruction. Probing was done and showed patent nasolacrimal duct, and mini Monoka stent was inserted through the right lower punctum, and the wall of the distended lower canaliculus was trimmed off and sutured over it. Inspection of the excised lesion was suggestive of cavernous hemangioma and the specimen was sent for histopathological examination. The patient tolerated the procedure well and was discharged the following day on topical medications (Fig. 1c). He shared the decision for the procedure and the necessity for follow up was explained.

On gross examination the lesion measured 2 × 1.8 × 0.4 cm. Cross sectioning revealed a solid and pale cut surface. Histopathologically, the conjunctival epithelium was thin and shows metaplasia with loss of goblet cells. The substantia propria showed diffuse infiltration mostly by mature neoplastic grade 1 cells resembling normal plasma cells with rare mitotic figures and few immature, binucleated and giant plasma cells (Fig. 1d). Immunohistochemical staining was done and showed expression of antibodies to CD38, CD 79a, CD138, and restricted kappa light chain (Fig. 1e, f, & 1g). The tumor cells were negative for lambda light chain staining, and did not express the following markers: IgG4, CD3, CD56 and CD20. This concluded the diagnosis of a conjunctival plasmacytoma.

MM work up was advised and the patient was referred to the hematology/oncology team for systemic workup. However, the patient was lost to follow up. He was approached by the treating team and his particular condition was fully explained. However, taking into consideration that he is far from his family, the patient elected to go back to his home country for further investigations. This case report has been prepared in compliance with the updated SCARE 2018 criteria [4]. A general written informed consent was taken from the patient, which includes permission for anonymous use of information and photos for the purpose of publication.

## 3 Discussion

Primary extramedullary plasmacytomas are generally rare and orbital involvement is even less likely. Solitary plasmacytoma can be found in patients who have a history of MM or in asymptomatic cases where the diagnosis is not yet established, as in our patient [1,3]. However, according to the National Comprehensive Cancer Network (NCCN), bony or extramedullary plasmacytoma (confirmed by biopsy) is one of the diagnostic criteria of MM and requires systemic surveillance and regular follow ups [5]. Thuro et al. evaluated 30 patients with orbital plasmacytoma where all patients had MM, the majority of which (63%) were known to have the disease and 37% were diagnosed immediately after the tissue diagnosis of orbital plasmacytoma [6]. Furthermore, conjunctival primary involvement by EMP is unusual and has been reported in very limited cases in the literature, some of which were not associated with MM [1,[7], [8], [9], [10]]. A review of the reported solitary conjunctival lesions is summarized in Table 1 including our case.

**Table 1:** Summary of the previously 5 reported conjunctival EMP cases and our current case.

Case #	Author	Age (y)	Gender	Location	Duration	Clinical features	Histopathologic findings	IHC stains expression	Treatment
1	Seddon JM^6^	63	M	Right lower palpebral conjunctiva	Several years	Reddish, firm, non-tender, adherent to lid tissue	Mature plasma cells with eccentric nuclei and abundant cytoplasm	IgM, IgG and lambda light chains	Mass Excision
2	Vyas MC^7^	22	F	Left lower fornix conjunctiva	1 year	Greyish pink, soft smooth non-lobulated, non-reducible and non-fixed painless swelling	Thin fibrous capsule, cellular plasma cells with no connective tissue	Not mentioned	Radiotherapy
3	Adkins JW^1^	66	M	Left lower fornix, inferior epi-bulbar area, plica semilunaris and superior fornix	Not known	Diffuse, red infiltration	Diffuse infiltrate of well-circumscribed plasma cells with eosinophilic cytoplasm	lambda light chains	Radiotherapy
4	Yumori JW^8^	33	M	Right temporal bulbar conjunctiva	Several years	Elevated mobile, faint pink, vascularized lesion with 2 feeder vessels	Monoclonal kappa plasmacytoma	CD138, kappa and lambda chains	Mass Excision
5	Jiang J^9^	17	F	Left lower fornix conjunctiva	2 months	Mobile, elastic, pink mass with smooth surface	Sheets of plasma cells, high proliferation, mitotic figures, binucleated plasma cells	CD138, IgG and kappa light chains	Radiotherapy
6	Current Case	44	M	Right lower palpebral conjunctiva	1 month	Reddish, painless, non-tender, smooth surface, soft and cystic	Round mature plasma cells, few immature plasma cells, Binucleated and giant plasma cells	CD138, CD38, CD79a and kappa light chains	Mass Excision

EMP: Extramedullary plasmacytoma, Y: year, M: male, F: Female, IHC: Immunohistochemical.

The demographics of patients with EMP orbital involvement are similar to the typical MM with an age ranging between 40 and 70 years of age and almost equal male to female ratio [3,6]. However, few cases of EMP were seen in younger individuals particularly the ones presenting with conjunctival lesions.

Grossly, the lymphoplasmacytic tumors appear soft with poor circumscribed borders, unlike our case which had well-defined lesion confused as a cavernous hemangioma. Under the microscope, large uniform round plasma cells with displaced nuclei and variable mitotic activity can be seen [11]. The histological grading (Grade I to grade III) depends on the maturity and degree of differentiation of the proliferating plasma cells and the frequency of the mitotic figures [12]. Immunohistochemistry is crucial in confirming the diagnosis by demonstrating monoclonal kappa or lambda light chains or heavy chains and expression of CD138, CD79a similar to our patient. The primary treatment of solitary EMP according to NCCN is radiation to the involved area with or without surgery [5]. A regular follow up with a 3–6 months interval is recommended with a surveillance systemic work up including complete blood count with differential and platelet count, serum chemistry for creatinine, albumin and corrected calcium. Additionally, serum quantitative immunoglobulins, serum protein electrophoresis, 24-h urine analysis for total protein and urine protein electrophoresis, in addition to serum free light chain assay. Bone marrow aspirate and biopsy, imaging such as whole-body MRI, low dose CT scan or PET/CT scan are advised as clinically indicated. Skeletal survey can be done as clinically indicated or annually [5].

## 4 Conclusion

Conjunctival primary EMP is very rare, yet should be considered in the differential diagnosis of conjunctival lesions. The lesion can be associated with MM thus systemic workup, follow up and patient counselling are recommended and are essential. Since this can be the first presentation of MM, related morbidity can be significantly reduced through early diagnosis and intervention.

## Patient consent

A general written informed consent was taken from the patient, which includes permission for anonymous use of information and photos for the purpose of publication.

## Provenance and peer review

Not commissioned, Editor review.

## Ethical approval

Case reports do not require Ethical approval in our institution. This case report was prepared in accordance with the ethical standards and the Helsinki Declaration. No trial of new drugs or therapy is applicable in this case.

A general written informed consent was taken from the patient, which includes permission for anonymous use of information and photos for the purpose of publication.

## Sources of funding

No funding was received.

## Author contribution

First author: Collection of data, literature search, and manuscript drafting.

Second author: Literature search and sharing in the manuscript drafting.

Third author: Histopathological examination and final tissue diagnosis.

Fourth author: Histopathological review of slides, taking images, and overall review for editing of the manuscript as the corresponding author.

Fifth author: Supervision and provision of clinical data and surgical images.

## Registration of research studies

This is a case report, which does not require a registry.

## Guarantor

Dr. Hind Manaa Alkatan.

## Acknowledgment

None.

## Declaration of competing interest

Authors do not have any conflict of interest related to this case report.
